# Persistence and space preemption explain species‐specific founder effects on the organization of marine sessile communities

**DOI:** 10.1002/ece3.3853

**Published:** 2018-02-23

**Authors:** Edson A. Vieira, Augusto A. V. Flores, Gustavo M. Dias

**Affiliations:** ^1^ Programa de Pós‐Graduação em Ecologia Instituto de Biologia Universidade Estadual de Campinas (UNICAMP) Campinas Brazil; ^2^ Centro de Biologia Marinha Universidade de São Paulo (USP) São Sebastião Brazil; ^3^ Centro de Ciências Naturais e Humanas Universidade Federal do ABC (UFABC) São Bernardo do Campo Brazil

**Keywords:** community assembly, competition, density‐dependence, diversity, historical contingency, succession

## Abstract

Community assembly may not follow predictable successional stages, with a large fraction of the species pool constituted by potential pioneering species and successful founders defined through lottery. In such systems, priority effects may be relevant in the determination of trajectories of developing communities and hence diversity and assemblage structure at later advanced states. In order to assess how different founder species may trigger variable community trajectories and structures, we conducted an experimental study using subtidal sessile assemblages as model. We manipulated the identity of functionally different founders and initial colony size (a proxy of the time lag before the arrival of later species), and followed trajectories. We did not observe any effects of colony size on response variables, suggesting that priority effects take place even when the time lag between the establishment of pioneering species and late colonizers is very short. Late community structure at experimental panels that started either with the colonial ascidian *Botrylloides nigrum*, or the arborescent bryozoan *Bugula neritina*, was similar to control panels allowed natural assembling. In spite of high potential for fast space domination, and hence negative priority effects, *B. nigrum* suffered high mortality and did not persist throughout succession. *Bugula neritina* provided complex physical microhabitats through conspecific clustering that have enhanced larval settlement of late species arrivals, but no apparent facilitation was observed. Differently, panels founded by the encrusting bryozoan *Schizoporella errata* led to different and less diverse communities compared to naturally assembled panels, evidencing strong negative priority effects through higher persistence and space preemption. *Schizoporella errata* founder colonies inhibited further conspecific settlement, which may greatly relax intraspecific competition, allowing resource allocation to colony growth and space domination, thus reducing the chances for the establishment of other species.

## 1 INTRODUCTION

The assembly of ecological communities is recognized today as a combined result of deterministic niche‐based mechanisms and neutral stochastic processes (Adler, HilleRisLambers, & Levine, 2007; Chase, 2007; Chase & Myers, 2011; Vellend, 2010). While species‐specific tolerance to environmental conditions and species interactions (e.g., competition and predation) may underlie convergent community dynamics at different habitat patches (Berlow, 1997; Caro, Navarrete, & Castilla, 2010; Louette, De Meester, & Declerck, 2008), several different studies have shown considerable temporal and spatial variation in community structure across sites prone to similar environmental conditions (Almany, 2004; De Meester, Vanoverbeke, Kilsdonk, & Urban, 2016; Edmunds, 2014; Hart, 1992; Klingbeil & Willig, 2016; Sutherland & Karlson, 1977; Trowbridge, 2007). Therefore, processes other than the ones above may play important, although overlooked, roles in the regulation of species assemblages (Fukami, 2015; Irving, Tanner, & McDonald, 2007). Historical contingencies caused by differences in immigration, birth, death, and local species extinctions have been recognized for a long period as important factors affecting the organization of many communities (Allen, VanDyke, & Cáceres, 2011; Chang & Marshall, 2017; De Meester et al., 2016; Fukami, 2015; Sutherland, 1974; Sutherland & Karlson, 1977). One class of such contingencies is referred to as priority effects, that is, the effects of early arriving species on the subsequent establishment of other species (Connell & Slatyer, 1977; De Meester et al., 2016; Fukami, 2015). Depending on the identity of first colonizers, species assemblages may evolve through different trajectories and develop to multiple stable states (Chase, 2003; Fukami & Nakajima, 2011; Jiang, Joshi, Flakes, & Jung, 2011; Osman, Munguia, & Zajac, 2010; Petraits & Dudgeon, 2015; Sutherland, 1974; Sutherland, 1978). Their history may thus have long‐lasting effects on species composition and abundance (Stier, Geange, Hanson, & Bolker, 2013; Weslien, Djupström, Schroeder, & Widenfalk, 2011), regulating the access to available resources (Blaustein & Margalit, 1996; Zuo, Li, Ma, & Callaway, 2016), productivity (Martin & Wilsey, 2012), energy flow, and nutrient cycling (Fukami et al., 2010). From the conservationist point of view, priority effects may even affect resistance to invasive species (De Meester et al., 2016; Dickson, Hopwood, & Wilsey, 2012; Stuble & Souza, 2016) and disturbance events (Symons & Arnott, 2014). Considering longer‐term effects, historical contingency may underlie patterns of genetic structure (Sefbom, Sassenhagen, Rengefors, & Godhe, 2016) and ultimately species evolution (De Meester et al., 2016; Fukami, Beaumont, Zhang, & Rainey, 2007). Priority effects are thus deeply linked to species coexistence and the maintenance of biodiversity (Adler et al., 2007; Chesson, 2000; Sutherland, 1974; Vellend, 2010).

While historical contingencies can restrict our ability to forecast patterns of species assembling (Dickie, Fukami, Wilkie, Allen, & Buchanan, 2012; Sutherland, 1974), life history traits of some early colonizers may help predicting the consequences of priority effects (Cifuentes, Krueger, Dumont, Lenz, & Thiel, 2010; Cleland, Esch, & McKinney, 2015; Sutherland, 1978). Depending on traits of the early colonizers, priority effects may occur either by negative or positive species interactions, largely dictated by inhibition or facilitation during early stages of the development of communities (Fukami, 2015; Gerla & Mooij, 2014; Weslien et al., 2011). The best known inhibitory effects occur through niche preemption, when early founders monopolize important resources that would otherwise be available to other species (De Meester et al., 2016; Fukami, 2015; Sutherland, 1978), or when first colonizers act as ecosystem engineers, modifying their habitat and preventing the establishment of other organisms (Bonnici, Evans, Borg, & Schembri, 2012; Jones, Lawton, & Shachak, 1994). Depending on intrinsic species traits, however, ecosystem engineering may otherwise play an opposite role and facilitate species arriving later (Fukami, 2015; Jones et al., 1994), for example, by providing tridimensional substrates and therefore increasing settlement grounds (Russ, 1980), or through the mitigation of abiotic stress by supplying more benign microhabitats (Jurgens & Gaylord, 2016; Perea & Gil, 2014; Vogt et al., 2014), ultimately leading to species coexistence and an increase of biodiversity.

The extent to which founders affect the trajectory of developing communities may depend on how conspecifics of early‐colonizer species interact. In the case of species with complex life cycles, the effects of adult cues to propagules or juveniles are particularly important, because they may deeply affect local abundance and spatial distribution patterns of individuals (Lara‐Romero, Cruz, Escribano‐Ávila, García‐Fernández, & Iriondo, 2016). Attraction through chemical cues may constitute an efficient way to promote aggregation in suitable habitats (Pawlik, 1992; Robinson, Larsen, & Kerr, 2011; Silva‐Filho, Bailez, & Viana‐Bailez, 2012), indirectly increasing the strength of density‐dependent regulation mechanisms of founder populations. Positive density‐dependent interactions include the mitigation of abiotic stress in crowding intertidal invertebrates (Jurgens & Gaylord, 2016; Minchinton, 1997), caterpillars (Klok & Chown, 1999), and plants (Vogt et al., 2014); enhanced reproduction, such as fruit dispersal in plants (Blendinger, Loiselle, & Blake, 2008) and fertilization in marine invertebrates (Kent, Hawkins, & Doncaster, 2003; Levitan, Sewell, & Chia, 1992) and terrestrial woodlice (Broly, Deneubourg, & Devigne, 2013); and diminishing of predation risk in invertebrates (Denno & Benrey, 1997; Turchin & Kareiva, 1989) and vertebrates (Blumstein & Daniel, 2003; Carrascal, Alonso, & Alonso, 1990). Negative interactions usually lie in some sort of intraspecific competition, which may reach unsustainable levels under conditions of very high‐population density (Branch, 1975; Chisholm & Muller‐Landau, 2011; Gerla & Mooij, 2014; Hart & Marshall, 2009; Robins & Reid, 1997). Therefore, the potential for propagule dispersal and the strength of intraspecific competitive interactions after establishment may ultimately have shaped the selection of chemical responses to conspecific cues, either positively or negatively. To establish such a link, there is a need to assess conspecific responses in light of species‐specific strategies on resource use and competitive hierarchy. In this sense, we could expect species in which individuals grow fast and tend to monopolize resources to repel conspecifics (and thus reduce intraspecific competition), and species in which isolated individuals are poor competitors, and rely on the attraction of conspecifics to form more resistant aggregations (Hart & Marshall, 2009; Porensky, Vaughn, & Young, 2012).

Careful experimental manipulation of founder assemblages, followed by the examination of community trajectories, may help understanding how priority effects modulate species diversity and assemblage structure at advanced successional states (Chang & Marshall, 2017; Cifuentes et al., 2010; Edmunds, 2014). Such methodology, however, has been used majorly for simple organisms, and effects measured in terms of individual counts for species that contributed the most to contrasts between treatments (Geange, Poulos, Stier, & McCormick, 2017; Irving et al., 2007; Jiang et al., 2011). Probably because manipulating the abundance of more complex organisms in natural conditions is more challenging, effects on natural assemblages dominated by clonal or colonial plants or animals are less documented (De Meester et al., 2016; Fukami, 2015). For decades, the construction of marine facilities, such as harbors and marinas, has promoted several modifications in coastal ecosystems, increasing the availability of hard substrata, which may support a diverse community of sessile organisms (Bulleri & Chapman, 2010). While some studies show a clear competition–colonization trade‐off, and thus, sessile community succession being determined by niche‐based processes (Buss and Jackson, 1979; Edwards and Stachowicz, 2010) suggested that nontransitive competitive relationships among species may explain alternative community states across or within habitats under similar conditions. Under these circumstances, priority effects may be important, because common species cannot be classified a priori as either early or late successional species in any obvious way, and because propagules of several species belonging to distinct functional groups may be available anytime. The importance of this historical contingency has been extensively discussed in descriptive studies (Osman, 1977; Sebens, 1986), but as long as we know this is one of the few studies addressing experimentally in the field how the identity and survivor of founder species affect community assembling and diversity. To do so, we triggered different history contingencies by manipulating both the identity of early colonizers and the time lag between the founder arrival and the subsequent establishment of other species (Urban & De Meester, 2009) in sessile communities from shallow subtidal artificial substrates. We expected to find (i) founder‐specific priority effects, which would be (ii) more intense when the time elapsed between first colonization and the arrival of later species arrivals was longer. We also predicted (iii) lower diversity of advanced assemblages first colonized by strong competitors for bare space.

## 2 MATERIALS AND METHODS

### 2.1 Study site

**Figure 1:**
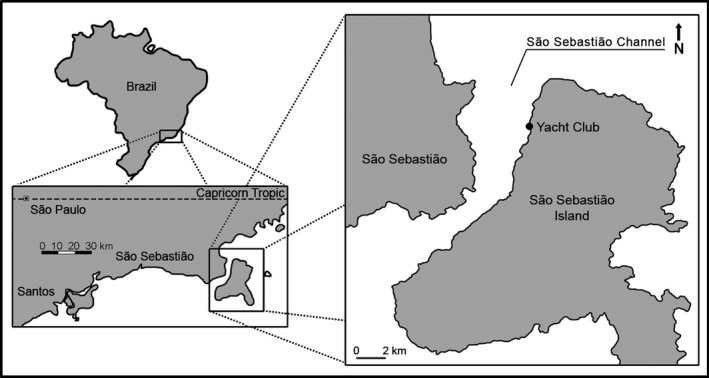
Yacht Club Ilhabela (YCI) study site at São Sebastião Island, southeastern Brazil (23°46′S/45°21′W). Modified from Vieira et al. (2016)

The study was carried out from April to September of 2013 at the Yacht Club Ilhabela (23°46′26.95″S/45°21′21.26″W), in the São Sebastião Channel, Southeastern Atlantic Coast of Brazil (Figure 1). This site is a recreational marina with a diverse fouling system growing on the sides of the floating blocks. Previous studies showed that the sessile community is dominated mostly by colonial ascidians and encrusting and arborescent bryozoans, but also sponges, barnacles, bivalves, polychaetes, and hydrozoans (Oricchio, Flores, & Dias, 2016; Oricchio, Pastro et al., 2016; Vieira, Dias, & Flores, 2016).

### 2.2 General experimental procedures

#### 2.2.1 Target founder species

We selected three invertebrate species that are common in the study area since early colonization to test how historical contingency related to variable colonization and eventual priority effects promoted by size advantage influences the development and structure of sessile communities: the flat soft‐bodied colonial ascidian *Botrylloides nigrum*, the flat calcified encrusting bryozoan *Schizoporella errata,* and the erect arborescent bryozoan *Bugula neritina*. Both flat founders are good space monopolizers (Kay & Keough, 1981; Nandakumar, 1996; Vieira, Duarte, & Dias, 2012; Vieira et al., 2016), but while the ascidian is less resistant to predation when not having an escape window that enables the colony to attain large sizes (Oricchio, Flores, & Dias, 2016; Osman & Whitlatch, 2004; Vieira et al., 2012, 2016), the hard mineralized body of the bryozoan confers resistance against predators since early stages (Lidgard, 2008). Conversely, the arborescent founder does not effectively monopolize space (Walters & Wethey, 1996) and could potentially facilitate the establishment of other organisms due to its tridimensional structure (Breitburg, 1985; Russ, 1980). Although we were not able to estimate all differences in life history traits among the species, we assumed that traits related to space preemption/provision and vulnerability to predation are of paramount importance to community dynamics, as long as space is a limiting resource. Previous observations in the same area showed that all the three above‐mentioned species are frequent during early stages (first 30 days) at experimental panels deployed year‐round, with *Schizoporella errata* being the most frequent species, occurring in 90% of all panels, followed by *Bugula neritina* in 80% and *Botrylloides nigrum* in 50% (Figure S1). Therefore, we assume that results from our experiments may generally reflect natural processes at the study area throughout the year.

#### 2.2.2 Priority effects through manipulation of initial size

Besides the manipulation of founder identity, we also tested the effects of founder size. This latter variable was used as a proxy of the time lag between first colonization and later species arrivals. Founders given more time to develop without the interference of other individuals would probably be prone to monopolize resources or modulate recruitment and survivor of later species by any other means. To test this idea, we started experimental panels with either younger small colonies (10–15 days old), given little advantage over later settling species, or older large colonies (20–30 days old), given large advantage over species coming next. Both time lags simulated natural stages during early colonization: The 10‐ to 15‐day lag represented a moment when bare space is still abundant and most of the colonies are still growing with no restriction imposed by border contacts with others, while the 20‐ to 30‐day lag represented a moment when more than 50% of the space is already covered, colonies may still find empty space to expand but contact interactions become common. Lags over 30 days were not considered as they would simulate an unrealistic scenario [i.e., colonies older than a month would hardly ever found isolated in nature (Dias, Delboni, & Duarte, 2008; Oricchio, Pastro et al., 2016; Vieira et al., 2012, 2016)]. Small and large ascidian colonies or encrusting bryozoans occupied an area around 0.5–1.0 and 1.5–2.0 cm^2^, respectively, while small and large erected bryozoan recruits bear 2–3 and 5–7 bifurcations, over an area of 1.0–1.5 and 3.0–3.5 mm^2^, respectively. Although the size of large colonies was near the double of small ones, the area they covered was still negligible compared to the overall area of experimental plates (around 5%, see below). Therefore, any effects of founding colony size would be an outcome of any advantages conferred to more advanced recruits (e.g., priority effects through improved conditions to resist environmental stress or predation, intra‐ or interspecific competition, etc.), and not because of differences in initial space preemption.

#### 2.2.3 Experimental setup

Inventory panels covered by sanded acetate sheets were deployed along the pier docks 2 months prior to the experiment in order to collect a sufficient supply of colonies of the target founder species within the needed size range. The selected settlers were then cut off from acetate sheets of inventory panels and glued with a cyanoacrylate adhesive on experimental sanded PVC panels (15.0 × 20.0 × 0.5 cm; Hart & Marshall, 2009). We cut the colonies off the acetate sheet keeping a free acetate border of as much or twice the size of the colony. This procedure was conducted underwater avoiding touching the colony and did not take longer than 2–3 min. To attach the acetate piece with the settled colony to the panel surface, we used a very small portion of glue, just sufficient to ensure attachment, avoiding leaking or contact with the colony. This needed to be a very fast procedure, no more than few seconds, to ensure no harm to colonies. Each experimental panel received four colonies of the same species and size around the center, leaving approximately 4 cm of distance among them. Twelve replicate panels were prepared for each combination of founder species (*B. nigrum*,* B. neritina,* and *S. errata*) and colony size (small and large, corresponding to the time lag between the foundation and the next arrival, i.e., 10–15 and 20–30 day, respectively) making up 72 panels. Treatment combinations were randomly interspersed along the docks, maintaining a distance of 2 m between neighboring panels. Panels were suspended at ~1.5 m depth, allowing a distance of 1.5 m from the sea bottom to avoid the negative impacts of suspended sediments for filter feeders. For each treatment combination, four panels were retrieved at 1 (early), 3 (mid), and 5 months (advanced). The early stage is characterized by plenty of bare space with no contact interactions among colonies and thus no competition for space. The mid stage is characterized by increased competition for space, with most colonies already contacting neighbor individuals, and the occurrence of both dead individuals and overgrowth. The advanced stage is characterized by almost no bare spaces and intense competitive interactions. At this stage, overgrowth by strong competitors was common.

In order to better describe the natural assembling and put into context any comparisons among experimental treatments, we deployed 12 extra sanded PVC panels over the same period without any interventions. These were also retrieved in three replicate groups (*n* = 4) at 1, 3, and 5 months after deployment.

### 2.3 Survival and coverage of founder colonies, and their effects on early recruitment

#### 2.3.1 Sampling

As persistence, space occupation, and density of founders can all affect community succession, we estimated survival rates and coverage for each founder species, besides testing whether their presence had an influence on early total and conspecific recruitment. For panels started with all founding species and colony sizes, we examined images obtained from experimental panels retrieved at 1 month to estimate (i) early survival of founding colonies (percentage of founding colonies alive), (ii) space preemption (as percentage coverage of surviving founder colonies, using a 100 intersection grid), (iii) the number of recruits of all fouling species, and (iv) the number of recruits of each tested founder species. For the latter, we assumed conspecific aggregation if more new colonies of species “a” were found in panels started with species “a,” compared to panels started with species “b” or “c.” The opposite result would indicate inhibition of conspecific.

#### 2.3.2 Data analyses

Survival data, coverage, total recruitment per panel and per available bare space were analyzed using a two‐way ANOVA, with fixed factors “founder identity” (three levels) and “colony size” (two levels). Conspecific recruitment rates were analyzed separately for each founder species, using the same orthogonal model. We also compared total recruitment per panel and per available bare space of experimental founder treatments (*B. nigrum*,* B. neritina*, and *S. errata*) with panels prone to natural assembling using one‐way ANOVA. “Colony size” was not included here because it did not apply to natural plates and also because it was not significant in any case. For all analyses, the Kolmogorov–Smirnov test was performed to check normality, and the Levene test was performed to check homogeneity of variances. Pairwise Tukey post hoc testing was used to further examine significant sources of variation.

### 2.4 Effects of founder identity and time lag on community assembly

#### 2.4.1 Sampling procedure

After 1 (early), 3 (mid), and 5 months (advanced stage), sets of four replicate panels of all seven treatments (the six combinations of species and colony sizes, plus a set of replicate panels exposed to natural assembling) were retrieved from the field, placed in coolers filled with sea water, and transported to the laboratory. All panels were scanned under a dissecting microscope to identify species to the lowest possible taxonomic level, and photographed to estimate the absolute percentage of area covered by the main taxa as a measure of abundance (following Vieira et al., 2016, 2012). Analyses were limited to the central 13 × 13 cm area to avoid marginal areas exposed to manipulation.

#### 2.4.2 Data analyses

Taxa richness estimates were compared using a three‐way ANOVA model, testing for “founder identity,” “colony size,” and “stage” (also a fixed factor, with three levels: early, mid, and advanced). As for total recruitment, we performed an additional analysis comparing richness of experimental founder treatments with panels exposed to natural assembling. Assumptions checking and post hoc comparisons were carried out as above.

The absolute cover area (%), an indicator of abundance of all the invertebrate groups identified, was used to compare community structure among founder treatments and over time, using the same model described above (i.e., testing the effects of “founder identity,” “colony size,” and “stage”). We built resemblance matrices using Bray–Curtis distances on square‐root transformed data, and performed respective PERMANOVA tests using 999 permutations (Anderson, 2001). PERMDISP tests for dispersion homogeneity were carried out for each successional stage (Anderson, 2001). Pairwise post hoc comparisons within significant sources of variation were used to test for specific contrasts. Species contributing the most to between‐group differences were identified using the SIMPER procedure (Clarke, 1993). We compared community structure of experimental founder panels with those left to natural assembling following the same multivariate procedures described above.

## 3 RESULTS

### 3.1 Survival and coverage of founder colonies, and their effects on early recruitment

#### 3.1.1 Survival and coverage

**Figure 2:**
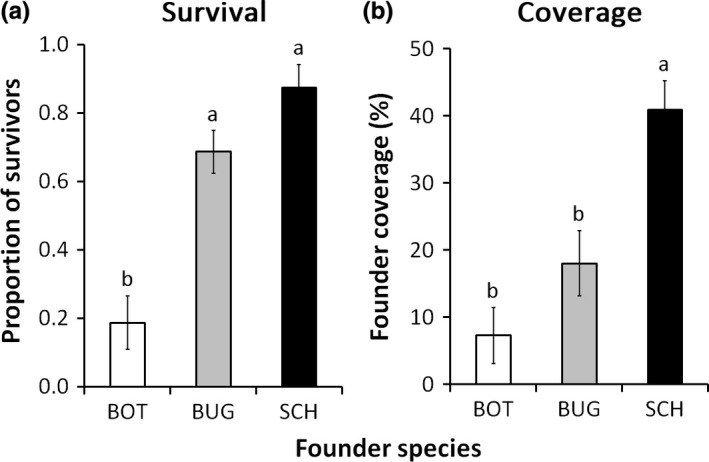
Mean proportion of survivors (±*SE*) and mean percentage coverage (±*SE*) of founder colonies of *Botrylloides nigrum* (BOT, white bars), *Bugula neritina* (BUG, gray bars), and *Schizoporella errata* (SCH, black bars) at early stage (1 month after the deployment of panels). Contrasts between groups labeled with different letters are significant (*p* < .05)

Regardless of colony size, both survival and coverage differed among founder species (Table 1). Colonies of the bryozoans *S. errata* and *B. neritina* survived more, respectively 90% and 70%, than colonies of *B. nigrum* which suffered 80% mortality after 30 days (Figure 2a). At that time, *S. errata* founders covered around 40% of the available space, which corresponded to the double of the area covered by *B. neritina* founders and five times the area covered by *B. nigrum* founders (Table 1, Figure 2b).

**Table 1:** Summary results of two‐way ANOVAs comparing the survival and coverage of small and large colonies of the founder species *Botrylloides nigrum*,* Bugula neritina,* and *Schizoporella errata* at early stage (1 month after the deployment of panels)

Source of variation	Survival (KS = 0.17, ns; L = 0.49, ns)				Coverage (KS = 0.18, a; L = 1.48, ns)		
	*df*	MS	*F*	*p*	MS	*F*	*p*
Founder	2	1.01	23.28	**<.001**	2,359	14.53	**<.001**
Colony size	1	0.01	0.24	.630	22	0.14	.717
Founder × colony size	2	0.01	0.24	.789	194	1.20	.326
Error	18	0.04			162		

KS, Kolmogorov–Smirnov test for normality; L, Levene's test for homogeneity of variance; ns, nonsignificant.

a *p* < .05.

Bold values stand for significant effects.

#### 3.1.2 Effects on recruitment

**Figure 3:**
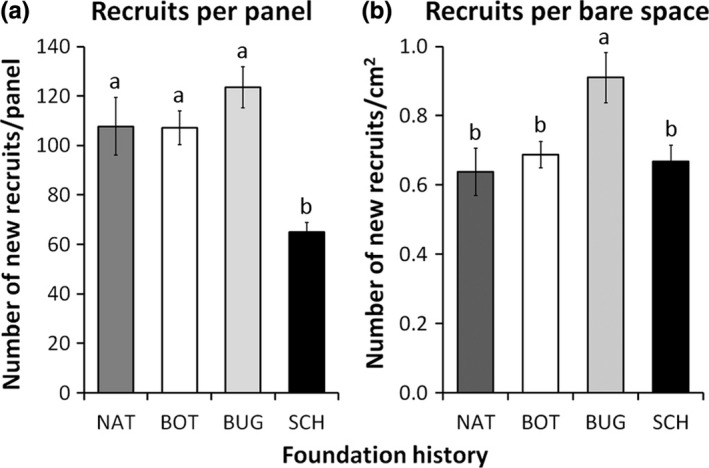
Mean number (±*SE*) of (a) total new recruits per panel (169 cm^2^) and (b) total new recruits per available bare space (cm^2^) at early stage (1 month after the deployment of panels) for panels founded naturally (NAT, dark gray bars) or experimentally by *Botrylloides nigrum* (BOT, white bars), *Bugula neritina* (BUG, light gray bars) and *Schizoporella errata* (SCH, black bars). For each recruit variable, contrasts between groups labeled with different letters are significant (*p* < .05)

Overall recruitment rates depended solely on the identity of founder species, with no detectable effects of initial colony size (Table 2, Figure 3). Panels founded by *S. errata* showed a lower number of total recruits when compared to panels founded by *B. nigrum* and *B. neritina* (Figure 3a). A joint analysis including the panels exposed to natural assembling (ANOVA: *F*_3,24_ = 13.38, *p* < .001) indicates that observations observed at panels founded by *B. nigrum* and *B. neritina* were aligned to the natural standard (Tukey test: *p* = .999 and *p* = .550, respectively), while the estimate obtained for *S. errata* panels was significantly lower (Tukey test: *p* < .05; Figure 3a). When adjusting estimates to account for the bare space still available on panels, recruit density (as individuals per cm^2^) was similar for *B. nigrum* and *S. errata* panels (Tukey test: *p* = .966) and lower compared to *B. neritina* panels (Tukey test: *p* = .032 and *p* = .019, respectively). When including the natural panels (ANOVA: *F*_3,24_ = 4.89, *p* = .009), recruit density at panels founded by *B. nigrum* and *S. errata* was aligned to standard conditions (Tukey test: *p* = .947 and *p* = .987, respectively), while *B. neritina* panels showed a higher number of new recruits per cm^2^ (Tukey test: *p* < .05 for all comparisons; Figure 3b).

**Table 2:** Summary results of two‐way ANOVAs comparing the number of total new recruits per panel and per available bare space in panels started with small and large colonies of *Botrylloides nigrum*,* Bugula neritina,* and *Schizoporella errata* at early stage (1 month after the deployment of panels)

Source of variation	Recruits per panel (KS = 0.12, ns; L = 1.64, ns)				Recruits per bare space (KS = 0.08, ns; L = 1.64, ns)		
	*df*	QM	*F*	*p*	QM	*F*	*p*
Founder	2	7295.17	21.8	**<.001**	0.145	5.66	**.012**
Colony size	1	80.67	0.2	.629	0.008	0.33	.573
Founder × colony size	2	585.17	1.7	.202	0.013	0.51	.607
Error	18	334.81			0.026		

KS, Kolmogorov–Smirnov test for normality; L, Levene's test for homogeneity of variance; ns, nonsignificant.

Bold values stand for significant effects.

**Figure 4:**
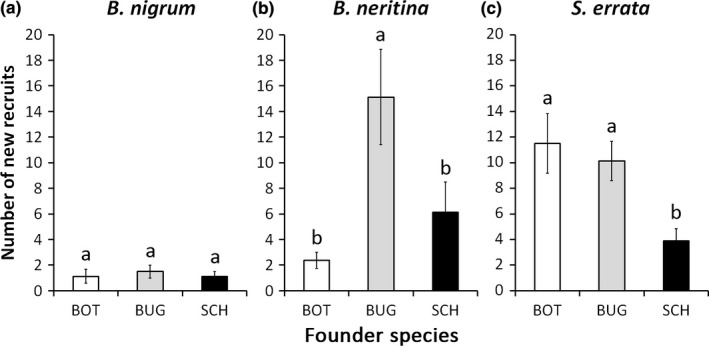
Mean number (a) *Botrylloides nigrum*, (b) *Bugula neritina,* and (c) *Schizoporella errata* new recruits at early stage (1 month after the deployment of panels) founded naturally (NAT, dark gray bars) or experimentally by *B. nigrum* (BOT, white bars), *B. neritina* (BUG, light gray bars) and *S. errata* (SCH, black bars). For each recruit variable, contrasts between groups labeled with different letters are significant (*p* < .05)

Effects of tested species on conspecific recruitment were quite different and also did not depend on colony size (Table 3, Figure 4a–c). *Botrylloides nigrum* recruited at very similar rates across treatments (Figure 4a). *Bugula neritina* apparently showed an aggregation pattern, as new recruits were nearly six and three times more abundant in *B. neritina* founded panels compared to the ones started with *B. nigrum* and *S. errata*, respectively (Figure 4b). Finally, *S. errata* likely inhibits the early recruitment of conspecifics nearby, as counts of *S. errata* new colonies were almost the triple on panels founded by *B. neritina* and *B. nigrum* than in communities first colonized by *S. errata* (Figure 4c).

**Table 3:** Summary results of two‐way ANOVAs comparing the number of new recruits of each tested founder species (estimate of conspecific aggregation/inhibition), in panels started with small and large colonies of *Botrylloides nigrum*,* Bugula neritina,* and *Schizoporella errata* at early stage (1 month after the deployment of panels)

Source of variation	*B. nigrum* recruits (KS = 0.17, ns; L = 0.85, ns)				*B. neritina* recruits (KS = 0.14, ns; L = 2.60, ns)			*S. errata* recruits (KS = 0.14, ns; L = 1.65, ns)		
	*df*	QM	*F*	*p*	MS	*F*	*p*	MS	*F*	*p*
Founder	2	0.38	0.2	.835	343.50	8.4	**.003**	132.13	5.4	**.014**
Colony size	1	0.17	0.1	.779	165.38	4.1	.060	24.00	1.0	.334
Founder × colony size	2	0.29	0.1	.869	100.50	5.5	.115	13.63	0.6	.581
Error	18	2.06			41.07			24.36		

KS, Kolmogorov–Smirnov test for normality; L, Levene's test for homogeneity of variance; ns, nonsignificant.

Bold values stand for significant effects.

### 3.2 Effects of founder identity and colony size on community assembly

#### 3.2.1 Taxa richness

**Figure 5:**
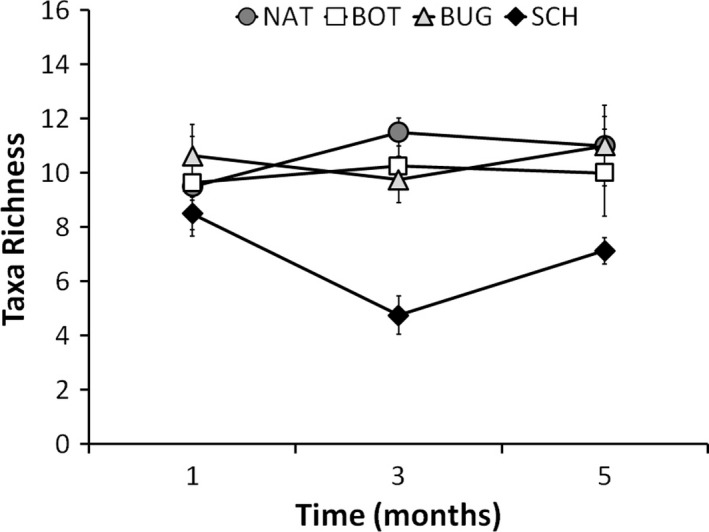
Mean taxa richness (±*SE*) at early, mid, and advanced stage (1, 3, and 5 months, respectively) for panels founded naturally (NAT, dark gray circles) and experimentally by *Botrylloides nigrum* (BOT, white squares), *Bugula neritina* (BUG, light gray triangles), and *Schizoporella errata* (SCH, black diamonds)

The number of taxa was variable over time and dependent on founder identity, but not colony size. Communities founded by *B. nigrum* and *B. neritina* supported remarkably similar richness (Tukey test: *p* = .904), around 10 species, during the whole experiment, while communities started by *S. errata* were on average substantially less speciose, with less than seven species (Tukey test: *p* < .001). General differences throughout the experiment were the result of a significant richness increase from months 3 to 5 (Tukey test: *p* = .010; Table 4). The number of species in control panels exposed to natural dynamics did not differ from richness estimates in communities founded by *B. nigrum* and *B. neritina* (Tukey test: *p* = .999 and *p* = .996, respectively), but was significantly higher than the number of species observed in panels founded by *S. errata* (Tukey test: *p* = .001; Table 5, Figure 5).

**Table 4:** Summary results of multifactorial ANOVA for taxa richness and PERMANOVA for community structure on panels founded by small and large colonies of *Botrylloides nigrum*,* Bugula neritina,* and *Schizoporella errata* over time (1, 3, and 5 months)

Source of variation	Taxa richness (KS = 0.10, ns; L = 1.06, ns)				Community structure (PD1 = 2.0, ns; PD3 = 2.1, ns; PD5 = 2.9, ns)		
	*df*	MS	*F*	*p*	MS	Pseudo‐ *F*	*p*
Founder	2	118.22	16.30	**<.001**	5,737	6.10	**.001**
Colony size	1	1.68	0.23	.632	573	0.61	.774
Stage	2	34.85	4.80	**.012**	15,843	16.84	**.001**
Founder × colony size	2	3.72	0.51	.602	650	0.69	.810
Colony size × stage	2	15.10	2.08	.135	996	1.06	.405
Founder × stage	4	14.95	2.06	.099	2,348	2.50	**.001**
Founder × stage × colony size	4	4.83	0.67	.619	1,166	1.24	.167
Error	54	7.26			941		

KS, Kolmogorov–Smirnov test for normality; L, Levene's test for homogeneity of variance; PD, PERMDISP test for homogeneity of dispersion (1, 1 month; 3, 3 months; 5, 5 months); ns, nonsignificant.

Bold values stand for significant effects.

**Table 5:** Summary results of two‐way ANOVA for taxa richness and PERMANOVA for community structure regarding foundation history (experimentally founded by *Botrylloides nigrum*,* Bugula neritina, and Schizoporella errata* or naturally founded) over time (1, 3, and 5 months)

Source of variation	Taxa richness (KS = 0.08, ns; L = 1.60, ns)				Community structure (PD_1m_ = 2.4, ns; PD_3m_ = 2.4, ns; PD_5m_ = 2.0, ns)		
	*df*	*MS*	*F*	*p*	*MS*	Pseudo‐*F*	*p*
History	3	84.78	11.73	**<.001**	5,189	4.67	**.001**
Stage	2	17.81	2.46	.092	18,007	16.20	**.001**
History × stage	6	14.02	1.94	.086	2,445	2.20	**.001**
Error	72	7.23			1,111		

KS, Kolmogorov–Smirnov test for normality; L, Levene's test for homogeneity of variance; PD, PERMDISP test for homogeneity of dispersion (1 m, 1 month; 3 m, 3 months; 5 m, 5 months); ns, nonsignificant.

Bold values stand for significant effects.

#### 3.2.2 Community structure

**Figure 6:**
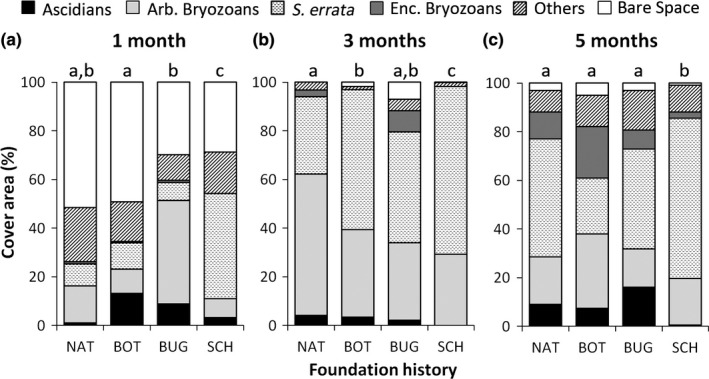
Cover area of main taxonomic groups (ascidians, arborescent bryozoans, encrusting bryozoans, and others—barnacles, bivalves, filamentous algae, hydrozoans, polychaetes, sponges) at (a) early (1 month), (b) mid (3 months), and (c) advanced (5 months) stages for panels founded naturally (NAT) and experimentally by *Botrylloides nigrum* (BOT), *Bugula neritina* (BUG) and *Schizoporella errata* (SCH). For each stage, differences between groups sharing a single letter are not significant (*p* > .05)

The differences in community structure among panels founded by different species were variable over time, regardless of colony size (Table 4). After 1 month, each founder species led to a different community structure (Tukey test; *p* < .05 for all pairwise‐comparisons). Panels founded by *B. nigrum* were dominated by ascidians, panels founded by *B. neritina* were dominated by arborescent bryozoans, and panels founded by *S. errata* were dominated by encrusting bryozoans (Figure 6a). The species that most contributed to these differences were the founders themselves and opportunistic species that colonize vacant space during colonization as hydrozoans and filamentous algae (Appendix: Table S1). After 3 months, differences in community structure were observed only between species assemblages founded by *B. nigrum* and *S. errata* (Pairwise‐comparison; *p* = .035), mostly because of a higher abundance of some arborescent bryozoan species in *B. nigrum* founded panels (Appendix: Table S1, Figure 6b). Communities started by *B. neritina* were not different from those founded by the two other species because of a shared domination of encrusting bryozoans. After 5 months, panels founded by *S. errata* were very different from the others (Pairwise‐comparisons; *B. nigrum* × *S*. *errata*:* p* = .001; *B. neritina* × *S*. *errata*:* p *= .001), basically due to a broad dominance of *S. errata* itself, in this treatment (Appendix: Table S1, Figure 6c), while other bryozoans, such *Electra tenella* and *Crisia pseudosolena* were more abundant on panels founded by *B. nigrum* and serpulids in panels founded by *B. neritina*. Overall, communities founded by *B. nigrum* and *B. neritina* were majorly similar to natural panels during the whole study, sharing around 10%–15% of ascidian coverage after 5 months, which was not the case of panels founded by *S. errata* that always differed (Table 5, Figure 6).

## 4 DISCUSSION

As predicted, our study indicates that the identity of founder species has an important role in the subsequent organization of sessile communities. However, contrasting to our predictions, significant departures from the natural assembling process, in which chances of priority effects were not increased by any means, were only evident for only one founding species, the space‐monopolizing species *Schizoporella errata*, regardless of initial colony size. When arriving first, *S. errata* decreased the chances of later species to settle nearby by preempting space, resulting in less diverse communities over time, majorly dominated by the founder itself. Differently, *B. neritina*, which do not monopolizes space, and *Botrylloides nigrum*, that is potentially able to fast space preemption but did not persist longer, led to similar richness and structure compared to natural untouched communities, restraining growth rates of clonal species and ensuring baseline species coexistence. Negative priority effects of *S. errata* were shown to be pervasive over time, possibly owing to this species resistance to predators (Oricchio, Pastro et al., 2016), and long colony duration (Sutherland, 1978; Sutherland & Karlson, 1977), and should only relax when aged colonies eventually detach, creating new space available for the establishment of other species.

Our results differ from those reported in other studies that suggest larger priority effects when the founding population is either numerous or constituted by larger individuals, ensuring more effective resource preemption (De Meester et al., 2016; Fukami, 2015; Poulos & McCormick, 2014). This mechanism is evident, for instance, when comparing exotic and native pioneering plants, with exotic species commonly showing strong priority effects owing to their higher ability of resource use, which ensures faster biomass accumulation both in terms of increasing individual numbers and size. Such effects are still important when well‐adapted native species settle shortly after pioneers (De Meester et al., 2016; Dickson et al., 2012). Similarly, large coral reef fishes exhibited stronger priority effects with only 3 hr of residence before the arrival of other fishes (Poulos & McCormick, 2014). We ensured a differential chance of advantage for arriving first by allowing founding colonies of two size categories (a proxy of time lag) to more (for long‐lag/large colonies) or less (for short‐lag/small colonies) relaxed competition for resources before the establishment of later species (Urban & De Meester, 2009). Although we expected that colonies of different sizes would show different potential of monopolizing or providing resources, we surprisingly did not observe differences on the extent of outcomes between the two lags tested. This means that priority effects, such as the ones found for *S. errata*, take place very fast, probably right after settlement (<15 days) when colonies are just a few zooids large.

In spite of its potential to rapidly spread over flat surfaces (Kay & Keough, 1981; Nandakumar, 1996), and thus to impose priority effects through resource preemption when arriving first (De Meester et al., 2016; Fukami, 2015), we observed no effect for the founding colonial ascidian *B. nigrum* on community structure. This species exhibited the lowest persistence, likely as a result of low‐survival rate and the lack of conspecific cues leading to colony clusters and thus the formation of larger and more resistant patches. Low resistance to predation, as previously observed for ascidians in the study area (Oricchio, Flores, & Dias, 2016; Vieira et al., 2012, 2016) and elsewhere (Jurgens, Freestone, Ruiz, & Torchin, 2017; Osman & Whitlatch, 2004) may have played an important role. As such, we conclude that potentially strong competitors, as *B. nigrum*, may impose very limited priority effects on community structure because they fail to persist under average environmental conditions. Differently, the encrusting bryozoan *S. errata* exerted strong priority effects through space preemption, deeply affecting recruitment and consequently community trajectory and structure, resulting in low diversity at advanced stages when compared to the other treatments, which showed abundant species other than the founders themselves covering larger areas. After 5 months, ascidians were virtually absent on panels founded by *S. errata* while covering 10%–15% of area in panels founded by *B. nigrum* and *B. neritina*. Besides, *S. errata* was the dominant encrusting bryozoan in *S. errata* founded panels, covering around 65% of the area, while panels founded by *B. nigrum* and *B. neritina* had other species contributing to the encrusting bryozoan coverage, with *Schizoporella* covering only around 20% and 40%, respectively. Fast growth led to rapid space monopolization, preventing the establishment of late‐arriving species close to *S. errata* colonies (Jackson & Hughes, 1985; Kay & Keough, 1981), which face a high risk of being dislodged or overgrown (Nandakumar, 1996; Russ, 1982). The high‐survival rate observed here coupled with the well‐known resistance against predators (Lidgard, 2008; Oricchio, Pastro et al., 2016) resulted in remarkable persistence of *S. errata* growing colonies, rendering pervasive effects on community structure, which may last until any disturbance event results in the detachment of aged colonies.

The capacity of *Schizoporella* to efficiently monopolize space and inhibit the arrival of other species was previously suggested in classic studies during the 70's, based on both observational and experimental approaches (Sutherland, 1978; Sutherland & Karlson, 1977). By forcing sets of experimental assemblages to be founded by functionally different sessile invertebrates, we provide here independent and novel evidence for the unique role of *Schizoporella* species worldwide in the determination of benthic community dynamics. In our study area, *S. errata* seems to be the strongest competitor in artificial hard substrates, owing both to its fast growth and resistance to predators (Vieira et al., 2016; Oricchio, Flores, & Dias, 2016; Oricchio, Pastro et al., 2016). Differently from earlier studies showing later *Schizoporella* die‐off and subsequent species replacement after 1 year (Sutherland, 1978; Sutherland & Karlson, 1977), high year‐round recruitment of *S. errata* likely allows this species to regain space and persist longer in the study area. Our results strongly suggest that large monospecific *Schizoporella* stands found on pier pilings at the Yacht Club of Ilhabela, and other boating facilities in the region (personal observations), may be primarily a result of priority effects, with selective predation on soft‐bodied invertebrates playing a complementary role.

We predicted that founders such as *B. neritina*, which does not monopolize resources but rather increase environmental complexity, could facilitate a large number of species, and therefore increase diversity when arriving first (Fukami, 2015). Our results do support overall settlement facilitation, and previous studies had indicated that intricate branches of *B. neritina* can provide settlers of different species valuable refuge from predation (Breitburg, 1985; Russ, 1980; Walters & Wethey, 1996). However, contrary to expectations, we found no priority effects in panels started with this species. In spite of creating physically complex substrates that could provide settlement secondary habitat and shelter from predators to an array of other species, the overall abundance of recruits in *B. neritina* plots did not differ from control ones. It is possible that gregarious settlement has led to exceedingly high levels of intraspecific competition (Chisholm & Muller‐Landau, 2011; Gerla & Mooij, 2014), ultimately decreasing founder persistence and thus any eventual long‐term positive effects on species diversity. Despite of showing a higher survival rate after 30 days when compared to *B. nigrum*,* B. neritina* did not persist in advanced community stages, allowing *S. errata* to colonize and occupy substantial space in panels. This nullified any possible priority effects, and shifted communities to states similar to the ones observed in naturally assembled panels.

Overall, our results highlight that priority effects caused by species capable of fast resource monopolization and that persist through time, such as *S. errata*, may change recruitment patterns and consequently further community assembly. *Schizoporella errata* remarkably precluded the establishment of several other species through space preemption, leading to relatively impoverished assemblages. While our results do not support avoidance as a mechanism underlying poor recruitment of the whole suite of species, they do strongly suggest the inhibition of conspecific recruitment near developing *S. errata* colonies. Such a response may relax intraspecific competition, allowing the allocation of resources to clonal growth resulting in fast resource monopolization. In contrast, poor competitors such as *B. neritina* may potentially create novel habitat for recruits of many different species, but still not cause longer‐term effects on community assembling because of their low persistence in the community. We conclude that founder effects ultimately depend on life history strategies of pioneering species, with density‐dependent effects on benthic stages playing a crucial role.

## DATA ACCESSIBILITY

Data are publically available at https://figshare.com/s/5ba40f6d523641ca8c80, https://doi.org/10.6084/m9.figshare.5313430

## CONFLICT OF INTEREST

None declared.

## AUTHORS’ CONTRIBUTIONS

EAV, AAVF, and GMD conceived the ideas and designed methodology. EAV collected the data. EAV, AAVF, and GMD analyzed the data. EAV, AAVF, and GMD wrote the manuscript. All authors contributed critically to early drafts and approved its submission for publication.
