# Supplementary material for: Persistence and space preemption explain species‐specific founder effects on the organization of marine sessile communities
Source: Ecol Evol. 2018 Feb 23;8(6):3430–42. doi: 10.1002/ece3.3853 (PMC5869360; doi:10.1002/ece3.3853)
Supplement: Supplementary file 1 [file ECE3-8-3430-s001.docx]

**Persistence and space preemption explain species-specific founder effects on the organization of marine sessile communities**

Edson A. Vieira^1¶*^, Augusto A. V. Flores^2^, Gustavo M. Dias^3^

^1^ Programa de Pós-Graduação em Ecologia, Instituto de Biologia, Universidade Estadual de Campinas (UNICAMP), CEP 13083-970, Campinas, SP, Brazil. ^2^ Centro de Biologia Marinha, Universidade de São Paulo (USP), CEP 11600-000, São Sebastião SP, Brazil. ^3^ Centro de Ciências Naturais e Humanas, Universidade Federal do ABC (UFABC), Rua Arcturus, 03 - Jardim Antares, CEP: 09606-070, São Bernardo do Campo, SP, Brazil. ^¶^ Current adress: Centro de Ciências Naturais e Humanas, Universidade Federal do ABC (UFABC), Rua Arcturus, 03 - Jardim Antares, CEP: 09606-070, São Bernardo do Campo, SP, Brazil. *Corresponding author: edson.vieira@ufabc.edu.br, +55 19 988266524

| **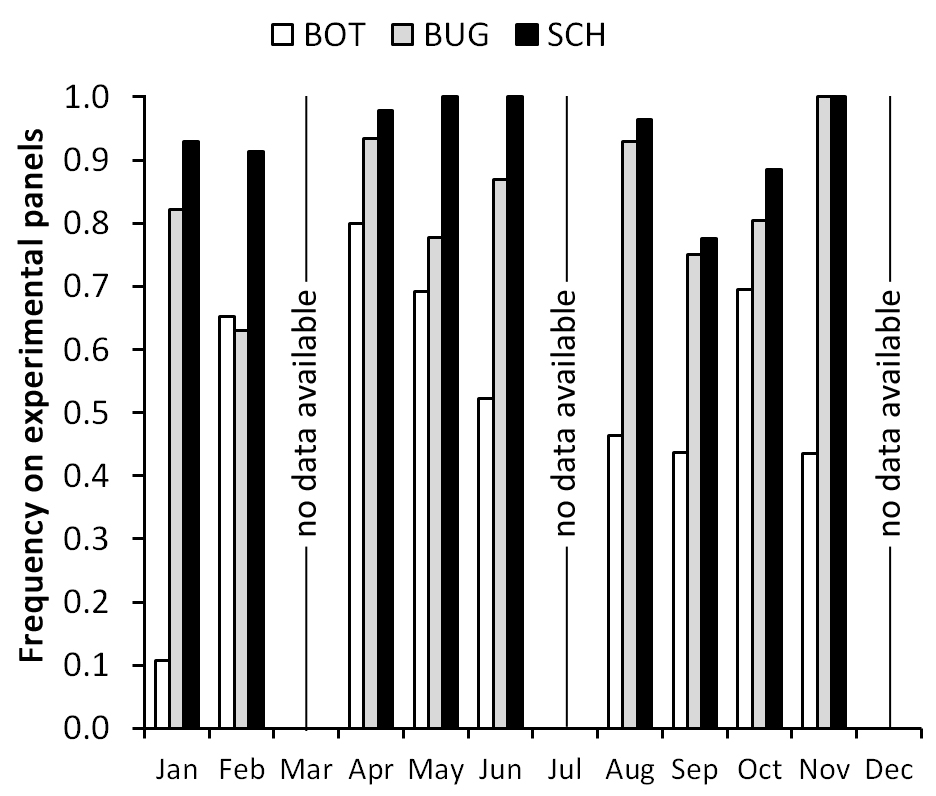** |
| --- |
| **Figure S1.** Year-round frequency of founder species *Botrylloides nigrum* (BOT), *Bugula neritina* (BUG) and *Schizoporella errata* (SCH) on experimental panels after 30 days of deployment (early stage of development). Data were gathered from studies conducted in the Yacht Club Ilhabela during 2010 (Sep and Oct), 2011 (Jan and Sept), 2013 (Apr and May), 2014 (Jun, Aug and Nov), 2015 (Oct) and 2016 (Feb and May). For months sampled twice (May, Sep and Oct) data represent means. |
