# Supplementary material for: Persistence and space preemption explain species‐specific founder effects on the organization of marine sessile communities
Source: Ecol Evol. 2018 Feb 23;8(6):3430–42. doi: 10.1002/ece3.3853 (PMC5869360; doi:10.1002/ece3.3853)
Supplement: Supplementary file 2 [file ECE3-8-3430-s002.docx]

**Persistence and space preemption explain species-specific founder effects on the organization of marine sessile communities**

Edson A. Vieira^1¶*^, Augusto A. V. Flores^2^, Gustavo M. Dias^3^

^1^ Programa de Pós-Graduação em Ecologia, Instituto de Biologia, Universidade Estadual de Campinas (UNICAMP), CEP 13083-970, Campinas, SP, Brazil. ^2^ Centro de Biologia Marinha, Universidade de São Paulo (USP), CEP 11600-000, São Sebastião SP, Brazil. ^3^ Centro de Ciências Naturais e Humanas, Universidade Federal do ABC (UFABC), Rua Arcturus, 03 - Jardim Antares, CEP: 09606-070, São Bernardo do Campo, SP, Brazil. ^¶^ Current adress: Centro de Ciências Naturais e Humanas, Universidade Federal do ABC (UFABC), Rua Arcturus, 03 - Jardim Antares, CEP: 09606-070, São Bernardo do Campo, SP, Brazil. *Corresponding author: edson.vieira@ufabc.edu.br, +55 19 988266524

**Table S1.** SIMPER results showing the relative contribution (%) of the three taxa that most contributed to significant pairwise differences between treatments founded by *Botrylloides nigrum* (BOT), *Bugula neritina* (BUG) or *Schizporella errata* (SCH), after 1 (early stage), 3 (mid stage) and 5 months (advanced stage) of deployment of panels. Abbreviations in front of each taxa stand for the major taxonomic group (AS – Ascidians, AG – Algae, AB – Arborescent Bryozoans, EB – Encrusting Bryozoans, H – Hydrozoans, P – Polychaetes) and the treatment in which the taxon was more abundant. ‘-’ Indicates cases in which the difference of treatment pairs was not significant in PERMANOVA test.

| Time |  | BOT x BUG | (%) |  | BOT x SCH | (%) |  | BUG x SCH | (%) |
| --- | --- | --- | --- | --- | --- | --- | --- | --- | --- |
|  |  |  |  |  |  |  |  |  |  |
| 1 month |  | *Bugula neritina* (AB, BUG) | 41.8 |  | *Schizoporella* *errata* (EB, SCH) | 42.7 |  | *Schizoporella* *errata* (EB, SCH) | 36.8 |
|  |  | *Obelia dicothoma* (H, BOT) | 10.8 |  | *Obelia dicothoma* (H, BOT) | 10.8 |  | *Bugula neritina* (AB, BUG) | 32.2 |
|  |  | *Botrylloides nigrum* (AS, BOT) | 10.2 |  | Filamentous algae (AG, SCH) | 9.9 |  | Filamentous algae (AG, SCH) | 7.7 |
|  |  |  |  |  |  |  |  |  |  |
| 3 months |  | - | - |  | *Schizoporella* *errata* (EB, SCH) | 28.2 |  | - | - |
|  |  | *-* | - |  | *Bugula neritina* (AB, SCH) | 24.0 |  | - | - |
|  |  | - | - |  | *Crisia* *peseudosolena* (AB, BOT) | 20.7 |  | - | - |
|  |  |  |  |  |  |  |  |  |  |
| 5 months |  | - | - |  | *Schizoporella errata* (EB, SCH) | 31.3 |  | *Schizoporella errata* (EB, SCH) | 29.3 |
|  |  | - | - |  | *Electra tenella* (EB, BOT) | 12.6 |  | *Bugula neritina* (AB, SCH) | 11.6 |
|  |  | - | - |  | *Crisia* *pseuodosolena* (AB, BOT) | 12.3 |  | Serpulids (P, BUG) | 7.6 |
